# Reply: The iron‐deficient rat as a model of restless legs syndrome: Was anything lost in translation?

**DOI:** 10.1002/mds.27263

**Published:** 2017-12-14

**Authors:** Yuan‐Yang Lai, Yu‐Hsuan Cheng, Kung‐Chiao Hsieh, Darian Nguyen, Keng‐Tee Chew, Lalini Ramanathan, Jerome M. Siegel

**Affiliations:** ^1^ Department of Psychiatry and Biobehavioral Sciences University of California Los Angeles California USA; ^2^ Veterans Administration Greater Los Angeles HealthCare System Sepulveda Los Angeles California USA

## Author Roles

(1) Research Project: A. Conception, B. Organization, C. Execution; (2) Statistical Analysis: A. Design, B. Execution, C. Review and Critique; (3) Manuscript Preparation: A. Writing of the First Draft, B. Review and Critique.

Y.‐Y.L.: 1A, 1B, 1C, 2A, 2C, 3A

Y.‐H.C.: 1C

K.‐C.H.: 2B

D.N.: 1C

K.‐T.C.: 1C

L.R.: 1C

J.M.S.: 1A, 3B

## Financial Disclosures

Y.‐Y.L. reports grant support from National Institutes of Health, NS082242. J.M.L. reports grant support from National Institutes of Health, DA034748.

We reported that iron‐deficient (ID) rats express signs of RLS.^1^ Silvani and colleagues^2^ suggested some critical issues for further study in the development of an animal model of RLS.

**Figure 1:**
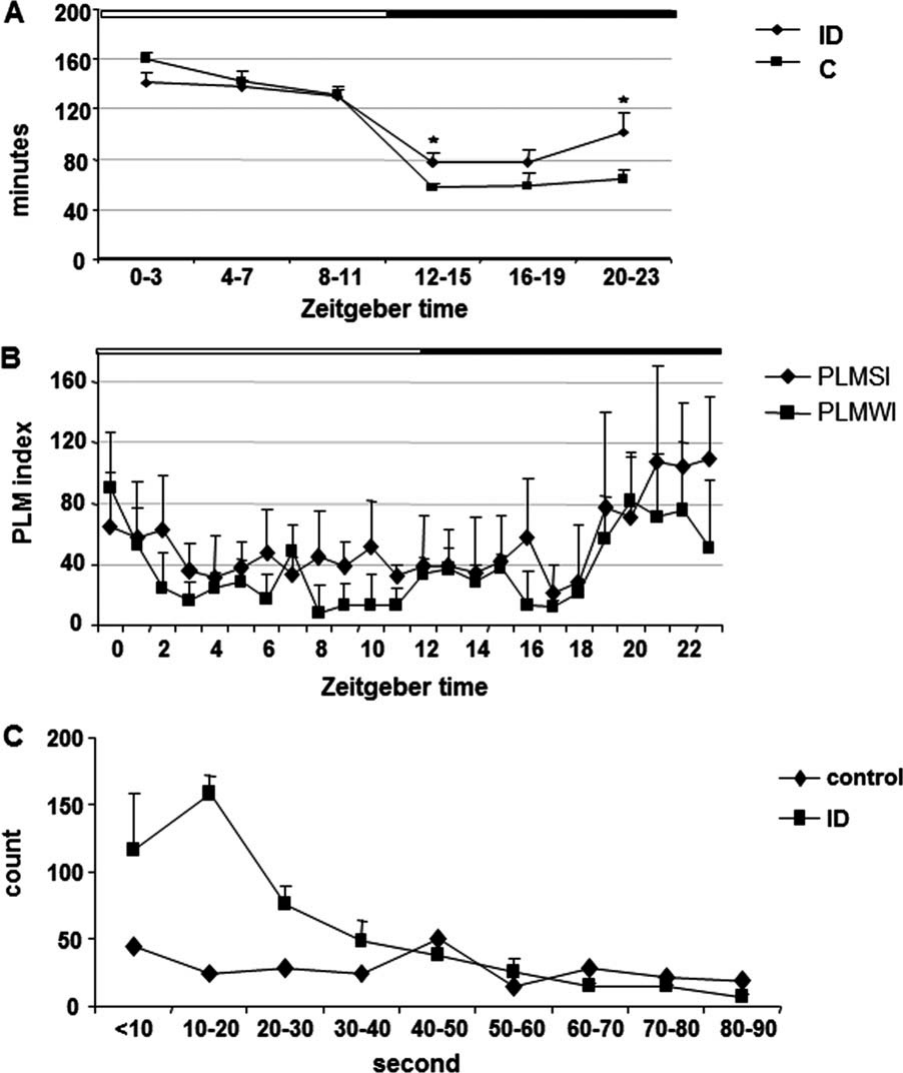
(A) Distribution of NREM sleep over 24‐hour recording in the control (C) and iron‐deficient (ID) rats. An increase in sleep time during the first and last 4 hours after lights off (active phase) was observed in the ID rats. The white and black bars shown on the top represent the light and dark phases, respectively. ^*^ *P* < 0.05, ANOVA; *df* = 11; n = 6 each control and ID rats. (B) Distribution of PLM index in quiet wake (PLMWI) and in sleep (PLMSI) over 24‐hour recording in the ID rats. PLMs, in both quiet wake and sleep, were maximal at Zeitgeber time (ZT) 19 to ZT23 and minimal at ZT2 to ZT18 (PLMWI) and ZT3 to ZT18 (PLMSI). Data are taken from the averaged recordings of 6 ID rats. The white and black bars shown on the top represent the light and dark phases, respectively; n = 6 each control and ID rats. (C) Distribution of IMIs of PLM in sleep in ID and control rats. Data were collected from 6 each of ID and control rats with 24‐hour sleep and motor recordings from each rat. The number of IMIs of the ID rat was the average of 6 rats, and the number of IMI of the control rat was obtained from 6 rats total. IMI was maximal at 10 to 20 seconds and minimal at 40 to 90 seconds in ID rats, whereas IMI in the control rat was equally distributed across all time intervals.

Data reported in our article were obtained from all animals, including 2 ID rats, which did not show periodic leg movements (PLM) in quiet wake (PLMW) and may not be considered as RLS‐like animals. Here, we reanalyzed the data excluding these 2 non‐RLS‐like ID rats and added 1 additional RLS‐like ID rat (6 each control and ID). The ID rats had significantly higher amount of sleep time during the dark phase than the control rats (Fig. 1), indicating excessive sleepiness in the active phase. A circadian pattern of motor activity was also found in the ID rats. PLMs in quiet wake and in sleep were increased and decreased starting 5 hours before and 3 hours after lights on, respectively (Fig. 1).

We reanalyzed interleg movement intervals (IMIs) using a 10‐second cutoff. Most IMIs in the ID rats fell between 10 and 20 seconds. In contrast, IMIs in the control rats tended to be equally distributed across all time intervals (Fig. 1). We calculated index PLMs in sleep (PLMSI) using the standard method (IMI >5 seconds) and the alternative method (IMI >10 seconds)^3^ and found that both methods adequately distinguished the RLS‐like pattern in these animals (ID; IMI >10 seconds: 48.1 ± 7.0, IMI >5 seconds: 52.8 ± 7.9; control; IMI >10 seconds: 6.7 ± 1.8, IMI >5 seconds: 7.4 ± 2.0).

Unlike humans, the sleep pattern in the rat is not a “perfect” diurnal circadian rhythm. Average bout duration of sleep and wake states in the rat are short, at 1.95 ± 0.35 minutes in wake and 1.67 ± 0.31 minutes in non‐rapid‐eye‐movement (NREM) sleep. This indicates that sleep‐wake‐state transition in rats is not stable. We adopted a criterion of 50% duration of EEG state in each 10‐second epoch. NREM sleep is scored if an epoch consisted of ≥50% of theta or lower frequency waves as well as behavioral observation on video recordings. We do not agree with Silvani and colleagues^2^ that this is in the “twilight zone” of the wake‐sleep transition. An increased EEG K‐alpha complex associated with PLMs is reported in RLS patients.^4^

Sleep posture in rodents is different from that in humans. The rat has to maintain a prone position in order to fall asleep and maintain sleep. Tonic contraction of the contralateral side of the leg is required to keep the animal in a prone position during leg movements, as seen in the figure.^1^ In addition, EEG alpha waves were observed during this period of recording, indicating a quiet wake state. Although increased EEG amplitude precedes leg movements observed in other examples of PLMW, the duration of the increased EEG amplitude did not exceed 50% of the 10‐second epoch.

The ID rats show excessive sleepiness in the active phase, circadian pattern of motor hyperactivity, and a similar pattern of IMI periodicity to human RLS. The ID rat may therefore be an ideal animal model of iron‐deficient anemia‐induced RLS.


Yuan‐Yang Lai, PhD,^1^
Yu‐Hsuan Cheng, MS,^1^
Kung‐Chiao Hsieh, PhD,^2^
Darian Nguyen, BS,^1^
Keng‐Tee Chew, MS,^1^
Lalini Ramanathan, PhD,^1^
Jerome M. Siegel, PhD^1,2^
*^1^Department of Psychiatry and Biobehavioral Sciences, University of California, Los Angeles, California, USA*
*^2^Veterans Administration Greater Los Angeles HealthCare System, Sepulveda, Los Angeles, California, USA*
